# Stem cell extracellular vesicles for neuropsychiatric disorders and translation

**DOI:** 10.20517/evcna.2026.14

**Published:** 2026-05-29

**Authors:** Hengxiang Fan, Shunqi Wang, Zhi Li, Tianze Yu, Chaolin Ma, Chong Sun

**Affiliations:** ^1^Department of Neurology, The Second Affiliated Hospital, Institute of Biomedical Innovation, Jiangxi Province Key Laboratory of Brain Science and Brain Health, and School of Basic Medical Sciences, Jiangxi Medical College, Nanchang University, Nanchang 330031, Jiangxi, China.; ^2^Queen Mary School, Jiangxi Medical College, Nanchang University, Nanchang 330031, Jiangxi, China.; ^#^These authors contributed equally to this work.

**Keywords:** Stem cell-derived extracellular vesicles, neuropsychiatric disorders, neuroinflammation, neuroregeneration

## Abstract

Neuropsychiatric disorders represent a major global health challenge due to their high prevalence, chronic disability, and substantial socioeconomic burden. Although stem cell-based therapies offer regenerative potential, their clinical application is limited by poor post-transplantation survival, restricted targeted integration, and potential tumorigenicity. Stem cell-derived extracellular vesicles (SC-EVs), particularly exosomes, have emerged as a promising cell-free therapeutic approach. These vesicles can cross the blood-brain barrier (BBB) and exhibit high biocompatibility and low immunogenicity. This review summarizes the cellular origins and biogenesis of SC-EVs and evaluates current preclinical and clinical evidence supporting their therapeutic potential. Particular attention is given to acute ischemic stroke and progressive neurodegenerative disorders, including Alzheimer’s disease and Parkinson’s disease. In addition, the molecular mechanisms underlying their neuroprotective and regenerative effects are discussed, with a focus on modulating neuroinflammation, promoting neurogenesis, and enhancing synaptic plasticity. Finally, key advances and major challenges in the clinical translation of SC-EVs are outlined. Integrating current evidence, this review provides a framework and practical perspective for the continued development of SC-EV-based therapies for complex neurological disorders.

## INTRODUCTION

Neurological disorders are a major global public health challenge, affecting approximately 3.4 billion people and causing nearly 11.8 million deaths annually, thereby imposing a heavy socioeconomic burden^[1]^. As the global population ages, the incidence of these disorders continues to rise, placing increasing pressure on healthcare systems^[2]^. A major obstacle to effective neurotherapeutics is the blood-brain barrier (BBB), which prevents most small molecules and nearly all macromolecular biologics from entering the central nervous system (CNS)^[3]^. In addition, neuronal damage in these disorders is complex, heterogeneous, and often irreversible, which limits the effectiveness of conventional monotherapies^[4]^. These challenges highlight the need for novel, multifaceted regenerative strategies.

Stem cells, characterized by self-renewal, proliferation, and multilineage differentiation, are considered promising candidates for the treatment of neurological disorders^[5,6]^. Common types include mesenchymal stem cells (MSCs), neural stem cells (NSCs), induced pluripotent stem cells (iPSCs), and embryonic stem cells (ESCs). These cells exert therapeutic effects through multiple mechanisms, including cell replacement, immunomodulation^[7]^, anti-inflammatory effects, promotion of angiogenesis, and enhancement of neuroprotection^[8]^. However, increasing evidence indicates that their therapeutic benefits are largely mediated by paracrine signaling rather than direct *in vivo* engraftment^[9]^.

Despite their therapeutic potential, current stem cell-based therapies face translational challenges. Intrinsic cellular heterogeneity complicates controlled differentiation and the standardization of clinical outcomes^[10]^. Moreover, poor post-transplantation survival and poor homing to injury sites limit efficacy, and there is a risk of immune rejection in allogeneic settings^[11]^. In addition, undifferentiated stem cells may form tumors, so careful safety measures such as rigorous purification and phenotypic validation are required before clinical use^[12]^.

In this context, stem cell-derived extracellular vesicles (SC-EVs) have emerged as a promising cell-free alternative. SC-EVs retain key regenerative and immunomodulatory components of their parent cells while avoiding many of the safety, immunological, and engraftment limitations associated with live-cell transplantation^[5,6]^. Although previous studies have summarized general aspects of extracellular vesicle biology, this review provides a focused and clinically oriented overview of SC-EV-mediated neurological repair, organized into six principal mechanistic categories: immunomodulation, mitochondrial homeostasis, neuroprotection, enhancement of neuroplasticity, angiogenesis, and clearance of pathological protein aggregates.

This review focuses primarily on conditions supported by strong preclinical and translational evidence, including acute ischemic injury (stroke) and progressive neurodegenerative disorders such as Alzheimer’s disease and Parkinson’s disease (PD). Emerging applications in neuropsychiatric disorders (e.g., epilepsy, schizophrenia, and major depressive disorder) are also discussed. These conditions are increasingly recognized not only as disorders of neurotransmission but also as involving structural and neuroinflammatory alterations in the CNS, making them relevant targets for SC-EV-based interventions. Finally, to bridge the gap between experimental research and clinical application, we evaluate the current clinical trial landscape and highlight key methodological challenges, including EV heterogeneity, isolation-related biases, and the lack of standardized characterization criteria that must be addressed to enable reliable clinical translation.

## LITERATURE SEARCH STRATEGY

**Figure 1:**
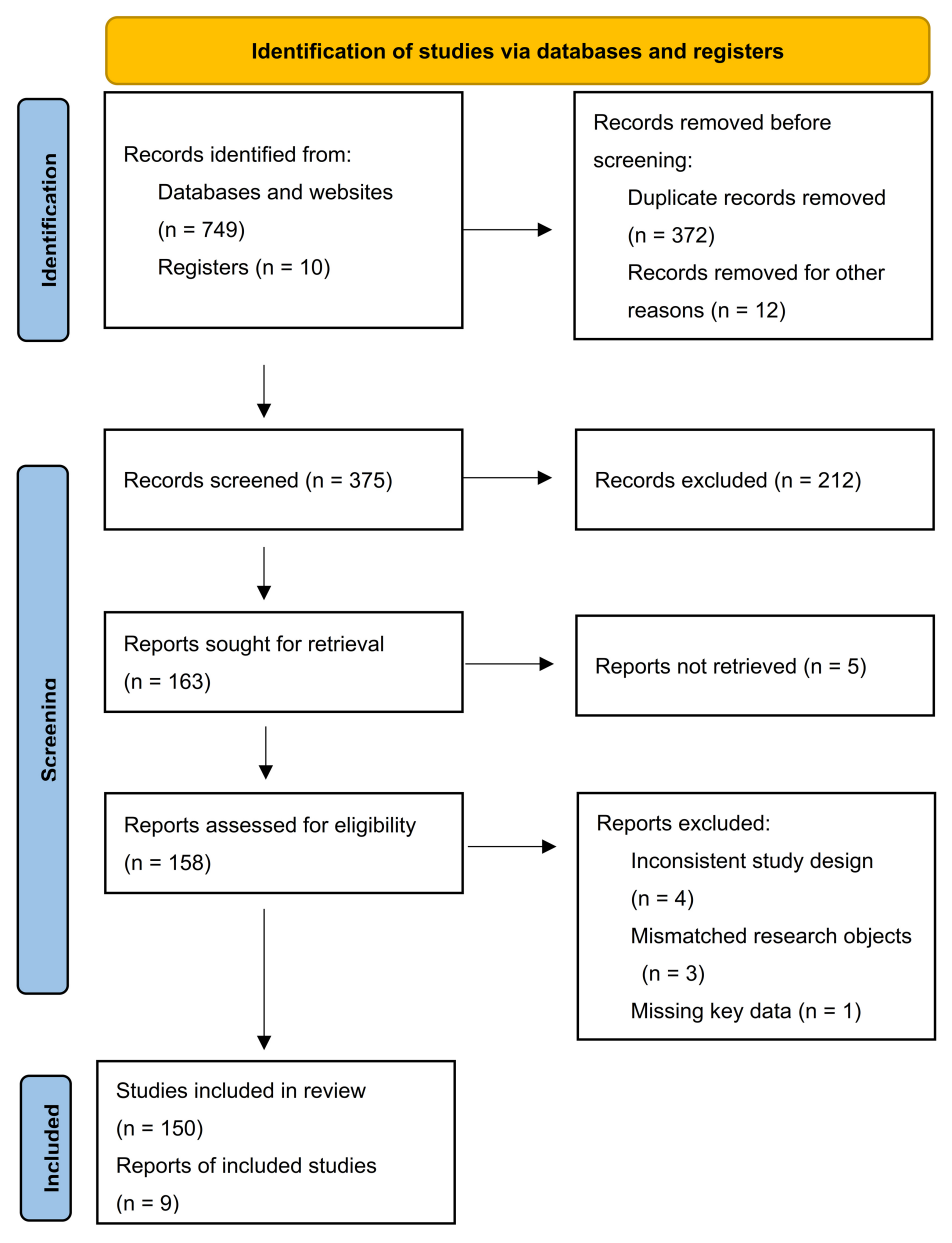
PRISMA flow diagram. This study followed the PRISMA 2020 guidelines for systematic literature screening. The initial search of databases and clinical trial registries yielded 759 records (749 from databases and websites and 10 from registries). After removing 372 duplicate records and 12 records excluded for other reasons, 375 records remained for screening. Title and abstract screening excluded 212 records that did not meet the inclusion criteria. Of the 163 records selected for full-text review, 5 could not be retrieved, leaving 158 articles for eligibility assessment. At this stage, 8 articles were excluded due to inconsistent study design, inappropriate study populations, or missing essential data. Ultimately, 150 records (148 journal articles and 2 registry entries) were included in final evidence synthesis. PRISMA: Preferred Reporting Items for Systematic reviews and Meta-Analyses.

A comprehensive literature search [Figure 1] was conducted to identify relevant studies on SC-EVs in neurological disorders. The major electronic databases, including PubMed, Web of Science, and Scopus, were systematically searched for articles published from 2015 to 2025. The search strategy combined keyword and Medical Subject Headings (MeSH) terms related to “stem cell-derived extracellular vesicles,” “isolation and purification,” and specific neurological disorders (e.g., stroke, epilepsy, schizophrenia, depression, neuropsychiatric diseases, neurodegenerative diseases).

Eligible studies included original research articles, preclinical studies, and clinical investigations that evaluated the therapeutic potential or underlying mechanisms of SC-EVs in neurological contexts. Review articles were also screened to identify additional relevant references. Studies not published in English or lacking sufficient methodological detail were excluded.

Two independent reviewers screened titles and abstracts for relevance, followed by full-text assessment of eligible articles. Discrepancies were resolved through discussion and consensus. Data extraction focused on study design, source of stem cells, EV isolation methods, experimental models, therapeutic outcomes, and mechanistic findings.

## SC-EVS: BIOGENESIS, SECRETION AND CELLULAR SOURCE

### Overview of SC-EVs

SC-EVs are nanoscale, membrane-bound vesicles (typically 30-150 nm in diameter) released by cells and involved in intercellular communication^[13]^. Their biological activity is mediated by cargo that includes proteins, lipids, and nucleic acids^[14]^. Proteomic analyses have identified enrichment of specific surface receptors, cytoskeletal components, and signaling molecules within these vesicles^[15]^.

According to the Minimal Information for Studies of Extracellular Vesicles (MISEV) guidelines^[16,17]^, the characterization of SC-EVs requires a multiparametric approach. Morphology and size distribution should be assessed using complementary high-resolution techniques, such as transmission electron microscopy (for morphology) and nanoparticle tracking analysis or tunable resistive pulse sensing (for size distribution and concentration). Quantification should include both total particle number and total protein content, as the particle-to-protein ratio provides an indirect measure of sample purity.

To confirm vesicle identity, protein profiling should demonstrate the presence of at least one transmembrane or lipid-associated marker (e.g., CD9, CD63, CD81) together with a cytosolic EV-associated protein [e.g., tumor susceptibility gene 101 (TSG101) or ALG-2-interacting protein X (Alix)]. In addition, at least one negative marker (e.g., calnexin for the endoplasmic reticulum or GM130 for the Golgi apparatus) should be evaluated to exclude contamination from intracellular organelles. Screening for common non-vesicular contaminants, including lipoproteins and protein aggregates, is also necessary to ensure that observed functional effects are attributable to EVs. Because no single method can fully define EV identity or purity, the combined use of multiple approaches is recommended^[17]^.

In addition to proteins, SC-EVs contain nucleic acids, including messenger ribonucleic acid (mRNA), micro ribonucleic acid (miRNA), and DNA fragments. Among these components, miRNAs are particularly important because they regulate gene expression and mediate intercellular communication. The miRNA composition of SC-EVs varies according to the cellular source, resulting in distinct regulatory profiles^[17,18]^. The lipid composition of SC-EVs also contributes to their biological activity. Enrichment of cholesterol, sphingolipids, and phosphatidylserine supports membrane stability and facilitates processes such as membrane fusion and endocytosis. Lipidomic studies further indicate that source-specific lipid profiles influence cargo loading and target cell interactions^[19-22]^.

### Biogenesis and release of SC-EVs

SC-EVs are formed through the maturation of multivesicular bodies (MVBs). During this process, the endosomal membrane invaginates to encapsulate selected cytosolic components, forming intraluminal vesicles (ILVs). These MVBs subsequently fuse with the plasma membrane, releasing ILVs into the extracellular space as vesicles enriched in tetraspanins such as CD63^[23]^.

The sorting and budding of vesicles are primarily regulated by the endosomal sorting complex required for transport (ESCRT) machinery. This system consists of four sub-complexes (ESCRT-0, -I, -II, and -III), which function sequentially to recognize ubiquitinated cargo, induce membrane invagination, and mediate vesicle scission. Accessory proteins such as hepatocyte growth factor-regulated tyrosine kinase substrate (HRS) and TSG101 facilitate cargo clustering, while VPS4 mediates membrane scission and complex disassembly^[24]^.

**Figure 2:**
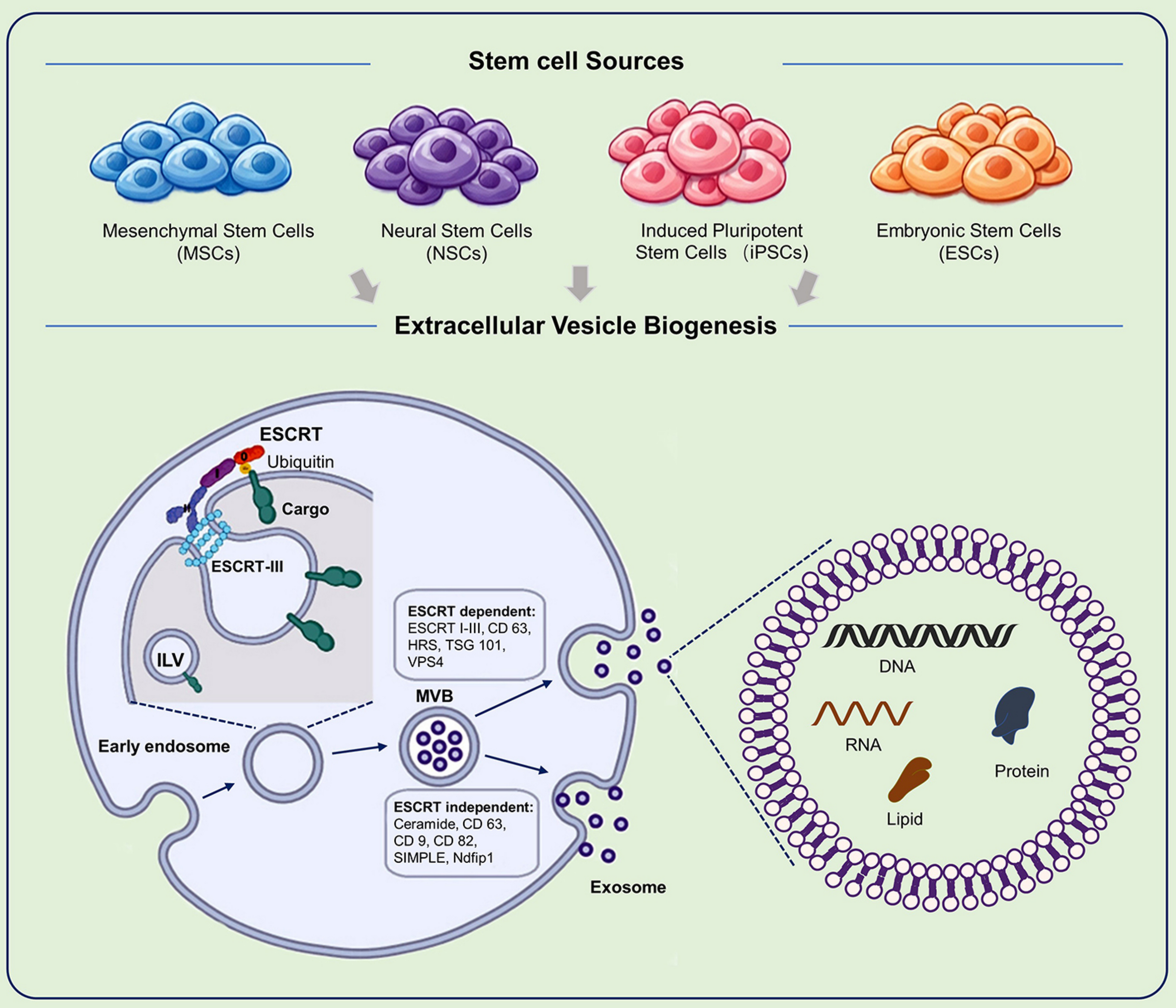
Biogenesis and Release of SC-EVs. This schematic illustrates the cellular pathways involved in the formation, maturation, and secretion of SC-EVs. The upper panel represents the parent stem cell, whereas the lower panel depicts key stages of vesicle biogenesis. The process begins with invagination of the plasma membrane to form early endosomes, which subsequently mature into late endosomes or MVBs. During this process, inward budding of the endosomal membrane encapsulates selected proteins, lipids, and nucleic acids within ILVs. Cargo sorting is regulated by both ESCRT-dependent and ESCRT-independent mechanisms. Upon maturation, MVBs fuse with the plasma membrane, releasing ILVs into the extracellular space as exosomes. In contrast, microvesicles are generated through direct outward budding and fission of the plasma membrane. SC-EVs: Stem cell-derived extracellular vesicles; ESCRT: endosomal sorting complex required for transport; HRS: hepatocyte growth factor-regulated tyrosine kinase substrate; TSG101: tumor susceptibility gene 101; VPS4: vacuolar protein sorting-associated protein 4; ILV: intraluminal vesicle; MVB: multivesicular body; SIMPLE：small integral membrane protein of the endosome; Ndfip1: Nedd4 family interacting protein 1; DNA: deoxyribonucleic acid; RNA: ribonucleic acid.

In addition to ESCRT-dependent pathways, SC-EVs can also be generated through ESCRT-independent mechanisms. One major pathway involves ceramide generation by neutral sphingomyelinases, which induces membrane curvature and promotes ILV formation. This process can be inhibited by neutral sphingomyelinase inhibitors such as GW4869. Tetraspanins (such as CD9, CD63, CD81) also contribute to cargo organization through membrane microdomains, independent of ubiquitination. Other proteins, including SIMPLE and Ndfip1, participate in cargo recruitment^[23]^. The coexistence of multiple biogenesis pathways within a single cell contributes to the heterogeneity of EV subpopulations [Figure 2].

### Cellular sources of SC-EVs

#### MSCs

MSCs are among the most extensively studied sources of therapeutic extracellular vesicles. Although MSC-derived EVs express canonical tetraspanins (CD9, CD63, and CD81), their secretomes exhibit substantial heterogeneity^[25-29]^, which affects their functional properties, as does methodological variation during isolation^[30-32]^.

Among MSC subtypes, bone marrow-derived MSCs (BMSCs) show strong neuroprotective potential but are limited by invasive harvesting procedures and batch variability^[15,33-37]^. In contrast, umbilical cord-derived MSCs (UCMSCs) provide a scalable and low-immunogenic alternative that is suitable for clinical applications^[36,38-42]^. Adipose-derived MSCs (ADMSCs) offer high yield through minimally invasive collection^[43-46]^; however, their isolation requires careful enzymatic processing to avoid altering EV composition^[15,47,48]^.

#### Pluripotent stem cells

Embryonic stem cell-derived EVs (ESC-EVs) contain potent regenerative cargo; however, their clinical use is limited by ethical concerns and the risk of teratoma formation^[49-55]^. iPSCs provide a more practical alternative for personalized therapy^[37,56]^. iPSC-derived EVs are enriched in developmental miRNAs and regulatory factors that promote angiogenesis and tissue repair. However, because their secretome can be highly dynamic, careful isolation and characterization are required to ensure consistency and reproducibility.

#### Neural stem and progenitor cells

Neural stem/progenitor cells (NSCs/NPCs) produce EVs that are suited for CNS repair^[57]^. These vesicles are enriched in neural-specific miRNAs and synapse-associated proteins^[58-60]^, supporting neuroprotection, anti-inflammatory effects, and axonal regeneration^[61,62]^. Large-scale production of NSC-derived EVs often relies on high-throughput methods such as Tangential flow filtration (TFF) for EV concentration; careful validation is required to preserve EV integrity and function.

### Isolation of SC-EVs

The isolation of SC-EVs from conditioned media remains a key technical challenge. Consistent with MISEV guidelines, no single method can be considered universally optimal; instead, each approach introduces specific biases that influence the composition of recovered EV populations^[63-65]^. Differential ultracentrifugation is widely used but may cause vesicle aggregation and co-isolation of contaminants such as protein complexes and lipoproteins. Size-exclusion chromatography (SEC) improves purity and preserves vesicle integrity; however, it often results in sample dilution and may require additional concentration steps^[64-66]^. Density gradient centrifugation, particularly with iodixanol, enables high-resolution separation based on buoyant density and reduces contamination from other similarly sized particles^[64,67]^.

Immunoaffinity capture using antibodies against surface markers (e.g., CD9 or CD63) allows selective enrichment of EV subpopulations but is limited by low throughput and potential interference with surface proteins^[64,68]^. Overall, the choice of isolation method influences EV composition and functional outcomes, thus highlighting the need for standardized characterization [Table 1].

**Table 1:** Methodological overview for stem cell-derived extracellular vesicle isolation

**Method/Technique**	**Principle**	**Key advantages**	**Major limitations**
Differential ultracentrifugation	Sequential centrifugation based on size/density sedimentation	Widely applicable^[69,70]^	Time-consuming; requires specialized equipment; Non-specific co-precipitation; vesicle damage/aggregation^[71-73]^
Size-exclusion chromatography	Separation by hydrodynamic volume using a porous column matrix	High purity; preserves vesicle integrity and function^[74,75]^	Moderate throughput; sample dilution^[17,76,77]^
Ultrafiltration	Size-based separation using membranes with defined molecular weight cut-offs	Scalable; suitable for processing large volumes^[78]^	Risk of vesicle deformation or membrane clogging^[79]^
Density gradient centrifugation	Separation based on buoyant density within a gradient medium	High-resolution separation from contaminants of similar sizes^[64,67,80]^	Labor-intensive; requires long centrifugation times^[81]^
Immunoaffinity capture	Selective binding using antibodies targeting specific surface markers	High specificity for distinct subpopulations or sources^[82]^	High cost; potential for antibody contamination; low throughput^[64,68,79]^

## PRECLINICAL RESEARCH OF EVS IN NEUROLOGICAL DISORDERS

### Immunomodulation and anti-neuroinflammation

The modulation of neuroinflammation is a core mechanism underlying the therapeutic effects of SC-EVs across neurological disorders. These effects involve regulation of glial activation states, suppression of pro-inflammatory mediators, and promotion of immune phenotypes associated with tissue repair.

#### Stroke: modulation of microglial dynamics and inflammatory cascades

In ischemic stroke, SC-EVs deliver molecular cargo that modulates inflammatory cascades and mitigates ischemia-reperfusion (I/R) injury. A key mechanism involves promoting a shift in microglia from a pro-inflammatory (M1-like) state toward a neuroprotective (M2-like) phenotype. For example, BMSC-EVs enriched in the lncRNA zinc finger antisense 1 (ZFAS1) reduce microglial activation by downregulating miR-15a-5p^[83]^, while other EV populations suppress the NLR family pyrin domain containing 3 (NLRP3) inflammasome to attenuate neuronal pyroptosis^[84]^. SC-EVs also influence post-transcriptional and post-translational regulation. BMSC-EVs carrying the lncRNA Krüppel-like factor 3 antisense RNA 1 (KLF3-AS1) inhibit sirtuin 1 (Sirt1) ubiquitination via USP22, alleviating I/R-induced injury^[85]^. In addition, UCMSC-EVs modulate microglial activity through high-mobility group box 1 (HMGB1)-mediated activation of the triggering receptor expressed on myeloid cells 1 (TREM1)-p38 mitogen-activated protein kinase (MAPK) pathway, whereas NSC-EVs deliver miR-125a-5p to inhibit the toll-like receptor 4 (TLR4)/nuclear factor-kappa B (NF-κB) signaling axis^[86-88]^. SC-EVs also reduce oxidative stress, for example by targeting the fat mass and obesity-associated protein (FTO)/N6-methyladenosine (m6A)/ dynamin-related protein (Drp1) pathway, which indirectly attenuates inflammation-associated neuronal injury^[89]^.

#### Neurodegenerative diseases: restoration of immune homeostasis and microglial balance

In Alzheimer’s disease (AD), SC-EVs reduce amyloid-β (Aβ) accumulation and associated glial activation, leading to decreased expression of pro-inflammatory cytokines such as tumor necrosis factor-alpha (TNF-α), interleukin-1 beta (IL-1β), and interleukin-6 (IL-6)^[90]^. In parallel, they increase brain-derived neurotrophic factor (BDNF) expression^[91]^ and promote a shift in microglial phenotype toward a reparative state. These changes may partially counteract age-associated inflammatory processes^[92,93]^ and reduce reactive astrogliosis^[94]^.

In PD, SC-EVs suppress inflammatory signaling in the substantia nigra, which is important for protecting dopaminergic neurons. Engineered BMSC-EVs (e.g., AK76 or quercetin-loaded EVs) promote M2-like polarization^[95]^, while Gli1-containing EVs inhibit Sp1-mediated activation of Leucine-rich repeat kinase 2 (LRRK2)^[96]^. UCMSC-EVs are preferentially internalized by activated microglia in lesion areas, contributing to localized immunomodulation and preservation of neuronal integrity^[97,98]^.

#### Emerging applications in neuropsychiatric disorders

In epilepsy, SC-EVs affect the interaction between neuroinflammation and neuronal hyperexcitability. ADMSC-EVs deliver miR-23b-3p to regulate the signal transducer and activator of transcription 1 (STAT1)/Glyoxylate reductase 1 homolog (GLYR1) axis and promote microglial phenotype transition^[99,100]^, while UCMSC-EVs activate nuclear factor erythroid 2-related factor 2 (NRF2) signaling to inhibit NLRP3 inflammasome activation^[101,102]^. iPSC-EVs also suppress inflammatory signaling via the lncRNA-0949-TLR4/MAPK/NF-κB pathway^[103]^.

In psychiatric disorder models, SC-EVs modulate inflammatory balance. In schizophrenia, MSC-EVs reduce pro-inflammatory cytokine expression while increasing IL-10 levels^[104]^. In depression models, UCMSC-EVs inhibit the myeloid differentiation primary response 88 (MyD88)/tumor necrosis factor receptor-associated factor 6 (TRAF6)/NF-κB pathway via miR-125b-5p, thereby reducing neuroinflammation and synaptic impairment^[105]^. ADMSC-EVs also activate adenosine monophosphate-activated protein kinase (AMPK)-dependent autophagy and suppress NLRP3 signaling^[106]^.

#### Critical appraisal and translational gaps

Although SC-EVs consistently attenuate neuroinflammation in preclinical studies, several limitations affect translational interpretation. First, many studies rely on the simplified M1/M2 polarization framework, whereas microglial activation *in vivo* represents a continuous and heterogeneous spectrum, including disease-associated microglia (DAM). Second, experimental models often focus on microglia in isolation, with limited consideration of interactions with astrocytes, endothelial cells, and peripheral immune components. Finally, variability in cytokine measurement and experimental design reduces cross-study comparability and reproducibility.

### Regulation of oxidative stress and mitochondrial homeostasis

Oxidative stress and mitochondrial dysfunction are common features of ischemic, neurodegenerative, and psychiatric disorders. SC-EVs reduce lipid peroxidation and support mitochondrial function through delivery of regulatory RNAs and proteins.

#### Stroke and PD: targeted modulation of redox status and mitophagy

In ischemic stroke, SC-EVs stabilize mitochondrial dynamics. For example, BMSC-EVs regulate the Krüppel-like factor 4 (KLF4)-FTO pathway and reduce the m6A modification of Drp1, thereby limiting mitochondrial fragmentation^[89]^. SC-EVs also reduce reactive oxygen species (ROS) production through mechanisms involving the lncRNA ZFAS1^[83]^ and phosphatidylinositol 3-kinase (PI3K)/protein kinase B (AKT)/mammalian target of rapamycin (mTOR) signaling^[88]^. In addition, NSC-EVs activate PINK1/Parkin-mediated mitophagy, promoting removal of damaged mitochondria^[107]^.

Similar mechanisms are observed in PD models. SC-EVs reduce oxidative stress in the substantia nigra and protect dopaminergic neurons. Both NSC-EVs^[98]^ and engineered BMSC-EVs^[95]^ decrease ROS levels and reduce neuronal loss in toxin-induced models.

#### Depression: antioxidant signaling and neuroprotection

Depression is increasingly associated with chronic oxidative stress and metabolic dysfunction alongside classical neurotransmitter imbalance. In this context, the therapeutic effects of SC-EVs are partly mediated by regulating redox homeostasis. BMSC-EVs increase hippocampal miR-26a levels and reduce oxidative damage markers, including malondialdehyde and nitric oxide^[108]^. In parallel, SC-EVs modulate downstream signaling pathways such as MAPK and NF-κB, thereby reducing apoptosis and supporting synaptic function^[109]^. These findings suggest that regulation of mitochondrial function and oxidative stress contributes to the neuroprotective effects of SC-EVs in depression models.

#### Critical appraisal and translational gaps

Despite consistent antioxidant effects in preclinical studies, several limitations remain. Most experimental models rely on acute oxidative stress paradigms that fail to reflect the chronic and progressive nature of mitochondrial dysfunction in human disease. In addition, the transient nature of ROS, together with variability in detection methods, complicates cross-study comparisons. The limited availability of *in vivo* tracking approaches makes it difficult to determine whether administered SC-EVs effectively reach target cells and interact with mitochondrial pathways.

### Neuronal protection and anti-apoptotic signaling

Programmed cell death is a common feature of both acute brain injury and chronic neurodegeneration. SC-EVs exert neuroprotective effects by modulating apoptotic pathways, reducing excitotoxicity, and preserving neuronal structure and function.

#### Stroke and AD: suppression of cell death pathways

In ischemic stroke, SC-EVs influence multiple forms of cell death. ADMSC-EVs deliver miR-25-3p to hypoxic neurons, suppressing p53 and the B-cell lymphoma 2 (BCL2)-interacting protein 3, thereby restoring autophagic balance and reducing apoptosis^[110]^. They also inhibit ferroptosis by targeting CHAC1 via miR-760-3p^[111]^. In addition, UCMSC-EVs regulate PI3K/AKT and MAPK signaling, decreasing pro-apoptotic factors (Bax, Caspase-3) while increasing anti-apoptotic Bcl-2 expression^[112]^.

In AD models, SC-EVs reduce amyloid-associated neurotoxicity and support neuronal survival. BMSC-EVs increase expression of neuronal and neurogenic markers [neuronal nuclei (NeuN), NeuroD1, BDNF]^[91,113,114]^, while UCMSC-EVs reduce Aβ burden and partially restore synaptic gene expression^[115]^. NSC-EVs also activate Sirt1 signaling to support mitochondrial function under amyloid stress^[94]^.

#### PD, epilepsy, and schizophrenia: targeted neuroprotection

In PD models, SC-EVs protect dopaminergic neurons through metabolic and autophagy-related mechanisms. Engineered BMSC-EVs (AK76 and quercetin) promote autophagy^[95]^, while EV-associated tumor necrosis factor stimulated gene 6 protein (TSG 6) reduces 1-methyl-4-phenylpyridinium toxicity via the STAT3/miR-7/neural precursor cell expressed developmentally down regulated protein 4 (NEDD4) pathway^[116]^. In epilepsy, EV-delivered circRNA hsa_circ_0000288 regulates cell cycle associated protein 1 (CAPRIN1)/glutamate receptor ionotropic NMDA subunit 3B (NR3B) signaling, reducing hippocampal damage^[117]^. ADMSC-EVs also inhibit NLRP3-mediated pyroptosis^[118]^. In schizophrenia models, MSC-EVs protect parvalbumin-positive interneurons and help maintain inhibitory circuit stability^[119]^.

#### Critical appraisal and translational gaps

Although SC-EVs reduce markers of apoptosis in preclinical studies, important limitations remain. Most models are based on acute injury paradigms that do not reflect the gradual and region-specific neuronal loss observed in human disease. In addition, many studies rely on short-term or static apoptosis markers, which may not predict long-term neuronal survival or functional integration. Therefore, it remains unclear whether these short-term protective effects translate into sustained functional recovery.

### Regulation of neurogenesis and synaptic plasticity

Impaired neurogenesis and synaptic dysfunction contribute to the progression of multiple neurological disorders. SC-EVs have been reported to promote neural progenitor proliferation, synaptic repair, and plasticity.

#### Schizophrenia and depression: neurogenesis and synaptic repair

In schizophrenia, MSC-EVs increase the number of doublecortin-positive cells in the hippocampus, suggesting reactivation of neurogenic pathways, potentially through restoration of BDNF signaling. They also upregulate the postsynaptic density protein 95 (PSD-95) and tyrosine hydroxylase, contributing to the stabilization of synaptic structure and dopaminergic neurotransmission^[104]^. Similar effects have been reported in depressive disorders. BMSC-EVs activate the BDNF/GDNF signaling axis and promote neuronal proliferation in the dentate gyrus (DG)^[120]^. NSC-EVs, particularly when combined with electroacupuncture, enhance synaptic protein expression, including PSD-95, synaptophysin (SYN), and growth-associated protein 43, thereby facilitating synaptic remodeling^[121]^. In addition, ADMSC-EVs inhibit Galectin-3-mediated microglial synaptic pruning, which may help preserve synaptic connectivity in the prefrontal cortex^[122]^.

#### AD and stroke: restoration of plasticity and network connectivity

In both neurodegenerative and ischemic conditions, SC-EVs contribute to the restoration of neural network integrity. In AD, NSC-EVs and UCMSC-EVs have been shown to partially normalize the expression of genes involved in synaptic plasticity^[94,115]^, which correlates with improved cognitive performance in amyloid precursor protein (APP)/presenilin 1 (PS1) models.

In stroke, SC-EVs support both neurogenesis and structural remodeling in peri-infarct regions. ADMSC-EVs engineered with PD-L1 and hepatocyte growth factor activate the STAT3/forkhead box O3 (FOXO3) pathway and promote neurogenesis^[123]^. In parallel, NSC-EVs enhance axonal structure and facilitate synaptic reconnection in the ischemic penumbra, contributing to functional recovery^[88,107]^. These effects are particularly relevant given that post-stroke recovery largely depends on the plasticity of surviving neural tissue.

#### Critical appraisal and translational gaps

Although SC-EVs promote neurogenesis and synaptic remodeling in preclinical models, significant translational challenges remain. Species differences in adult neurogenesis limit direct extrapolation to humans. In addition, it remains unclear whether EV-induced neurons and newly formed synapses can achieve stable, long-term functional integration within existing neural circuits *in vivo*.

### Angiogenesis and BBB integrity

Vascular dysfunction and BBB disruption are key features of both acute ischemic injury and chronic neurodegeneration. Rather than acting solely on neurons, SC-EVs also influence the neurovascular unit by promoting angiogenesis and stabilizing BBB structure.

#### Stroke: revascularization and barrier defense

In ischemic stroke, therapeutic strategies must balance reperfusion with the risk of secondary injury caused by BBB disruption. SC-EVs contribute to both processes. BMSC-EVs, particularly under hypoxic preconditioning, carry pro-angiogenic microRNAs such as miR-126-3p and miR-140-5p, which enhance endothelial cell migration and tube formation^[124]^. This response is further supported by increased expression of angiogenic factors, including vascular endothelial growth factor (VEGF), vascular endothelial growth factor receptor 2 (VEGFR2), angiopoietin-1 (Ang-1), tyrosine kinase with immunoglobulin and epidermal growth factor homology domains 2 (Tie-2)^[125]^.

In addition, SC-EVs help maintain BBB integrity. BMSC-EVs inhibit Caveolin-1-mediated internalization of tight junction proteins such as zonula occludens-1 (ZO-1) and claudin-5, thereby preserving endothelial barrier structure^[126]^. They also improve endothelial cell survival under hypoxic conditions through activation of PI3K/AKT and MAPK signaling pathways^[122]^.

#### AD: BBB repair and therapeutic delivery

In AD, BBB disruption contributes to disease progression by allowing peripheral inflammatory factors to enter the CNS. SC-EVs can mitigate this process. NSC-EVs have been reported to reduce BBB permeability and partially restore barrier function^[127]^. UCMSC-EVs suppress the TLR4/NF-κB signaling pathway via miR-125b-5p and provide vascular protection^[128]^.

#### Critical appraisal and translational gaps

Although SC-EVs show vascular protective effects in preclinical studies, current models often rely on acute injury paradigms and simplified leakage assays. These approaches do not fully capture the complexity of chronic human vascular dysfunction. In particular, it remains uncertain whether SC-EVs can restore long-term neurovascular coupling and physiological transport across the BBB.

### Regulation of pathological protein aggregation

The accumulation and propagation of misfolded proteins, including Aβ in AD and α-synuclein (α-Syn) in PD, are central to neurodegeneration. SC-EVs influence protein homeostasis through multiple mechanisms, including reducing protein production, enhancing degradation, and promoting clearance.

#### AD: clearance of Aβ via enzymatic degradation and phagocytosis

In AD, SC-EVs modulate several steps of the amyloid cascade. BMSC-EVs deliver miR-29c-3p, which suppresses β-secretase 1 (BACE1) and reduces the amyloidogenic APP processing^[129]^. They also promote Aβ clearance by activating the sphingosine kinase (SphK)/sphingosine-1-phosphate (S1P) pathway^[114]^ and increasing the expression of amyloid-degrading enzymes such as neprilysin (NEP) and insulin-degrading enzyme (IDE)^[113,130]^. In addition, UCMSC-EVs enhance microglial phagocytosis of Aβ deposits, contributing to reduced plaque burden^[92,115,131]^.

#### PD: attenuation of α-Syn aggregation and toxicity

In PD, SC-EVs reduce α-Syn accumulation and associated toxicity. Neural-differentiated ADMSC-EVs decrease intracellular α-Syn levels and may limit downstream neuroinflammatory responses^[132]^. NSC-EVs similarly reduce α-Syn burden in PD models^[133]^. These effects may alleviate lysosomal stress and help preserve dopaminergic neuron function.

#### Critical appraisal and translational gaps

Despite promising findings, current models often rely on artificial overexpression of mutant proteins, which does not fully reflect human disease progression. In addition, many studies focus on end-stage plaque measurements rather than dynamic processes such as oligomer formation and propagation. Therefore, it remains unclear whether SC-EVs can effectively halt long-term protein aggregation and disease progression *in vivo*.

### Summary of therapeutic mechanisms: the pleiotropic synergy of SC-EVs

**Figure 3:**
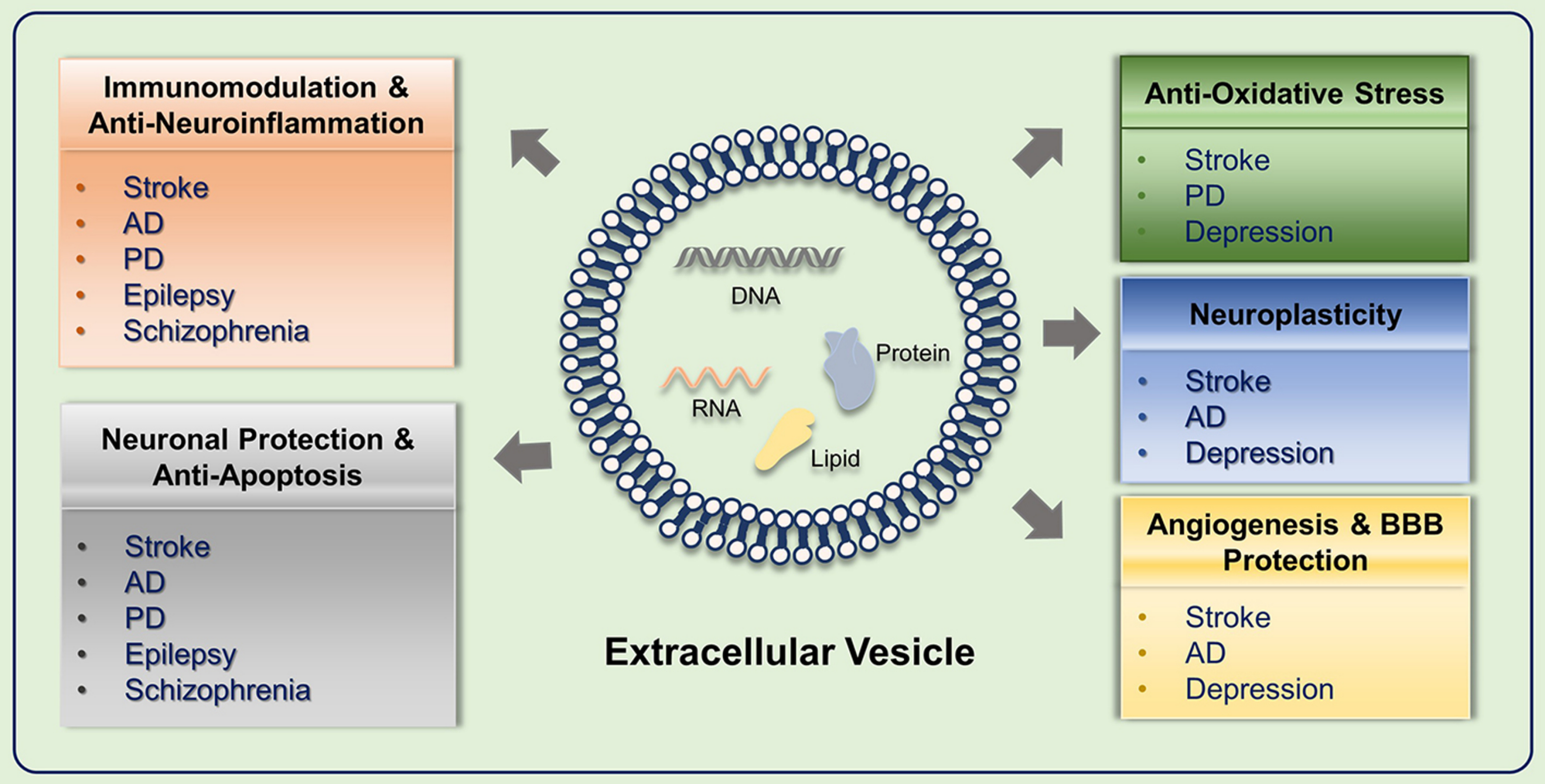
Integrated therapeutic mechanisms of SC-EVs in neurological disorders. This schematic illustrates six major functional pathways: (1) Regulation of Pathological Protein Aggregation to restore proteostasis in AD and PD; (2) Immunomodulation and Anti-Neuroinflammation alongside (3) Neuronal Protection and Anti-Apoptosis, which collectively provide broad cellular defense across acute injuries (stroke), chronic neurodegeneration (AD, PD), and psychiatric/seizure disorders (epilepsy, schizophrenia); (4) Anti-Oxidative Stress responses to restore intracellular redox balance in stroke, PD, and depression; (5) Neuroplasticity enhancement to rebuild synaptic circuitry; and (6) Angiogenesis and BBB Protection to repair the neurovascular unit in stroke and AD. SC-EVs: Stem cell-derived extracellular vesicles; AD: Alzheimer’s disease; PD: Parkinson’s disease; BBB: blood-brain barrier.

SC-EVs exert therapeutic effects through multiple coordinated mechanisms rather than a single target pathway. They deliver a combination of regulatory RNAs and proteins that influence inflammation, apoptosis, oxidative stress, synaptic function, and vascular integrity [Figure 3].

Unlike conventional pharmacological agents, EVs represent a cell-free system that retains key functional properties of stem cells while reducing safety concerns associated with cell transplantation. The molecular mediators underlying these effects are summarized in Table 2.

**Table 2:** Summary of SC-EV therapeutic mechanisms and targets in neurological disorders

**Therapeutic mechanism**	**Cellular origin of SC-EVs**	**Key cargo**	**Target/Pathway**	**Disease type**	**Ref.**
Immunomodulation and anti-neuroinflammation	BMSC	lncRNA ZFAS1	miR-15a-5p	Stroke	[83]
	BMSC	Data unavailable	NLRP3	Stroke	[84]
	UCMSC	Data unavailable	MyD88/TRAF6/NF-κB	Depression	[105]
	ADMSC	Data unavailable	AMPK-mTOR	Depression	[106]
	UCMSC	Data unavailable	NRF2	AD	[134]
Oxidative stress regulation	NSC	Data unavailable	PINK1/Parkin	Stroke	[107]
	ADMSC	miR-23b-3p	STAT1/GlyR1	Epilepsy	[99]
	BMSC	miR-146a-5p	TRAF6	Epilepsy	[135]
	UCMSC	NRF2, NLRP3	NRF2/NF-κB/NLRP3	Epilepsy	[101]
Neuronal protection	BMSC	lncRNA KLF3-AS1	miR-206/USP22	Stroke	[85]
	UCMSC	TSG-6	STAT3-miR-7-NEDD4	PD	[116]
	BMSC	Data unavailable	FTO siRNA; ATM mRNA	PD	[136]
	BMSC	Data unavailable	miR-26a	Depression	[108]
	BMSC	Data unavailable	BDNF	AD	[91]
	MSC	Data unavailable	BDNF	Schizophrenia	[104]
Neurogenesis promotion	BMSC	Data unavailable	miR-21-5p	Stroke	[125]
	UCMSC	Data unavailable	BDNF	Depression	[137]
Synaptogenesis and neuroplasticity regulation	ADMSC	Data unavailable	Galectin-3	Depression	[122]
	BMSC	circRNA hsa_circ_0000288	Caprin1	Epilepsy	[117]
Angiogenesis promotion	BMSC	Data unavailable	miR-126-3p; miR-140-5p; let-7c-5p	Stroke	[124]
Abnormal protein aggregation regulation	BMSC	Data unavailable	miR-29c-3p/BACE1; Wnt/β-catenin	AD	[129]
	UCMSC	Data unavailable	NEP; miR-211-5p	AD	[131]
	BMSC	Data unavailable	IDE	AD	[113]
	BMSC	Data unavailable	SphK/S1P	AD	[114]

SC-EVs: Stem cell-derived extracellular vesicles; BMSC: bone marrow-derived mesenchymal stem cell; UCMSC: umbilical cord-derived mesenchymal stem cell; ADMSC: adipose-derived mesenchymal stem cell; MSC: mesenchymal stem cell; NSC: neural stem cell; lncRNA: long non-coding RNA; ZFAS1: zinc finger antisense 1; miR: microRNA; NLRP3: NOD-like receptor family pyrin domain containing 3; MyD88: myeloid differentiation primary response 88; TRAF6: TNF receptor-associated factor 6; NF-κB: nuclear factor kappa B; AMPK: AMP-activated protein kinase; mTOR: mechanistic target of rapamycin; AD: Alzheimer’s disease; PINK1: PTEN-induced kinase 1; GlyR1: glycine receptor 1; STAT1: signal transducer and activator of transcription 1; NRF2: nuclear factor erythroid 2-related factor 2; KLF3-AS1: KLF3 antisense RNA 1; USP22: ubiquitin-specific peptidase 22; TSG-6: TNF-stimulated gene 6; STAT3: signal transducer and activator of transcription 3; NEDD4: neural precursor cell expressed developmentally down-regulated protein 4; PD: Parkinson’s disease; FTO: fat mass and obesity-associated protein; siRNA: small interfering RNA; ATM: ataxia telangiectasia mutated; mRNA: messenger RNA; BDNF: brain-derived neurotrophic factor; circRNA: circular RNA; Caprin1: cell cycle-associated protein 1; BACE1: β-site amyloid precursor protein cleaving enzyme 1; NEP: neprilysin; IDE: insulin-degrading enzyme; SphK: sphingosine kinase; S1P: sphingosine-1-phosphate.

### Clinical translation of EV therapy in neurological disorders

SC-EVs have attracted increasing attention in clinical research. Although preclinical studies provide substantial mechanistic evidence, clinical translation remains at an early stage. Most trials are ongoing, and only a limited number have reported completed outcomes^[138]^ [Table 3].

**Table 3:** Overview of clinical trials involving SC-EVs for neurological disorders

**Disease/Condition**	**NCT Number**	**Sample size**	**Cellular origin of SC-EVs**	**Dosing regimen**	**Phase**	**Endpoints**	**Reported outcomes/Notes**
Ischemic stroke	NCT06612710	69	Human-induced neural stem cells	Patients received intravenous injection of EVs at a dosage of 4 × 10^9^ particles/kg once daily for 7 days	Phase 1	Assess the safety; MRI; Scale scores	Data unavailable
	NCT07232563	9	hUC-MSCs	Patients received hUC-MSC-sEV-001 via intranasal instillation at a dosage of 1 × 10^11^ particles per administration. The treatment was initiated on the day of enrollment and continued daily for 4 days	Phase 1	Dose-Limiting Toxicity; Adverse events; Scale scores	Data unavailable
Acute ischemic stroke	NCT06995625	18	Wharton’s Jelly MSCs	EVs were administered once daily for 5 days. There were three cohorts, each with 3 to 6 participants, who received escalating doses: 4.8 × 10^10^, 9.6 × 10^10^, and 19.2 × 10^10^ particles, respectively	Phase 1	Assess the safety; Maximum tolerated dose; Assess the efficacy	Evidence from non-human primate models underscored the potential for functional neurological restoration
	NCT07145294	40	iPSCs	EVs (2 mL, 2 × 10^10^ particles/mL) were administered intranasally twice weekly for 12 weeks, in combination with standard-of-care treatment for cerebrovascular disease	Phase 2	Scale scores; Inflammatory indicators; Blood-brain barrier disruption indicators; Neuronal damage indicators	Data unavailable
AD	NCT04388982	9	Allogeneic adipose MSCs	Participants were assigned to three dosage groups: low-dose (5 μg), medium-dose (10 μg), and high-dose (20 μg). All groups received intranasal instillations (1 mL) twice weekly for 12 weeks	Phase 2	Assess the safety; Cognitive function; Quality of life evaluation; MRI Neuroimaging; Changes of AD biomarkers	The treatment was found to be safe and well-tolerated. Notably, the medium-dose group showed potential cognitive improvements and reduced hippocampal volume loss
PD	NCT05152394	20	Allogeneic adult umbilical cord MSCs	Participants received two doses of EVs via intranasal instillation on consecutive days. Each dose consisted of 4 mL of solution containing approximately 8 × 10^11^ exosomes	Phase 1	Assess the safety	Data unavailable
Refractory focal epilepsy	NCT05886205	34	iPSCs	Participants were assigned to four cohorts: a low-dose group (2 μg, *n* = 8), a mid-dose group (6 μg, *n* = 8), a high-dose group (18 μg, *n* = 8), and a dose-expansion group (*n* = 10). All participants received iPSC-Exos (200 μL) via nasal drip twice daily for 12 weeks	Early phase 1	Assess the safety; Seizure frequency	Data unavailable
Neurodegenerative diseases	NCT06607900	120	Human umbilical cord MSCs	Intranasal administration	Phase 1	Scale scores	Data unavailable

EV characterization and QC data are excluded from this table as they are not routinely reported in clinical trial registries prior to formal publication. SC-EVs: Stem cell-derived extracellular vesicles; NCT: National Clinical Trial; hUC-MSCs: human umbilical cord mesenchymal stem cells; MSC: mesenchymal stem cell; sEV: small extracellular vesicle; EV: extracellular vesicle; iPSC: induced pluripotent stem cell; AD: Alzheimer’s disease; PD: Parkinson’s disease; MRI: magnetic resonance imaging.

A completed clinical trial (NCT04388982) evaluated intranasal administration of allogeneic adipose-derived MSC-EVs (ahaMSC-EVs) in patients with mild-to-moderate AD. Participants received treatment twice weekly for 12 weeks across three dose groups. The intranasal route is of particular interest because it enables delivery to the CNS via olfactory and trigeminal pathways, thereby bypassing the BBB. The study demonstrated that repeated administration of ahaMSC-EVs was safe and well tolerated. The medium-dose group showed greater improvement in cognitive function and reduced hippocampal atrophy compared with the other groups^[139]^. These findings suggest that EV efficacy may not increase linearly with dose, and they highlight the need for optimized dosing strategies and pharmacokinetic characterization in future studies.

### Challenges and bottlenecks in clinical translation

Despite the recognized advantages of SC-EVs, including low immunogenicity and the ability to cross the BBB, their clinical translation remains limited by several challenges. These include technical constraints in production, regulatory complexity, and economic considerations.

### Production standardization and scalability

A major barrier to clinical application is the lack of standardized Good Manufacturing Practice (GMP)-compliant protocols for EV production and characterization. EVs are highly sensitive to the cellular microenvironment; therefore, variations in stem cell source, passage number, and culture conditions (e.g., bioreactor shear stress) can alter EV composition and subpopulation profiles. This variability contributes to inconsistencies in bioactive cargo, including miRNAs and proteins, and compromises reproducibility across batches. In addition, despite the development of MISEV guidelines, standardized and high-throughput methods for particle quantification and surface marker characterization (e.g., CD63, CD81) are still lacking, which further limits comparability between studies^[17,140]^.

### Dosage optimization and efficacy validation

Defining the clinically relevant dosing strategy remains a key pharmacokinetic challenge. Current preclinical studies use heterogeneous dosing regimens, and EVs do not follow conventional small-molecule pharmacokinetics. As a result, dose extrapolation from animal models to humans using standard allometric scaling is unreliable. Moreover, most clinical studies remain exploratory, with limited sample sizes, heterogeneous endpoints, and insufficient long-term follow-up, making it difficult to assess the sustained therapeutic effects^[141,142]^.

### Long-term safety and pharmacokinetics

Long-term biodistribution and safety data for SC-EVs remain limited. Although EVs are thought to preferentially accumulate at sites of injury or inflammation, systemically administered EVs are rapidly cleared by the mononuclear phagocyte system. A substantial proportion accumulates in off-target organs, particularly the liver and spleen. The long-term consequences of this peripheral accumulation, as well as the potential for immune responses following repeated administration, have not yet been fully characterized and require further investigation^[143-145]^.

### Regulatory and economic constraints

The regulatory framework for EV-based therapies is still evolving and varies across regions. Differences in classification criteria between agencies such as the US FDA and the European Medicines Agency (EMA) can lead to divergent approval pathways, particularly for engineered or modified EV products^[146,147]^. In addition, production costs remain high due to the need for controlled cell culture systems, advanced purification technologies, and comprehensive quality control. Current estimates suggest that EV-based therapies are substantially more expensive than conventional pharmaceuticals^[148,149]^. However, comprehensive health economic evaluations are still lacking.

These challenges are interrelated and will require coordinated efforts to address. Advances in scalable production technologies, improved standardization, and greater regulatory alignment are needed. Ultimately, well-designed, large-scale clinical trials will be necessary to establish the safety and efficacy of SC-EV-based therapies.

## CONCLUSION

SC-EVs represent a promising approach for the treatment of neurological disorders, owing to their ability to cross the BBB, deliver bioactive cargo, and modulate multiple pathological pathways. Future research should prioritize improving targeting efficiency, optimizing cargo loading, and evaluating combination approaches with existing therapies. EV-associated molecules may also serve as valuable biomarkers for disease monitoring and treatment response^[150]^. To support clinical translation, standard manufacturing protocols, well-defined dosing strategies, and reliable delivery systems must be established. Addressing these issues will be essential for moving SC-EVs-based therapies from experimental models toward clinical application.

## DECLARATIONS

### Copyright

© The Author(s) 2026.
